#  Effectiveness of Vitamin D Supplement Therapy in Chronic Stable Schizophrenic Male Patients: A Randomized Controlled Trial

**Published:** 2016

**Authors:** Fatemeh Sheikhmoonesi, Mehran Zarghami, Shima Mamashli, Jamshid Yazdani Charati, Romina Hamzehpour, Samineh Fattahi, Rahil Azadbakht, Zahra Kashi, Shahram Ala, Mona Moshayedi, Habibollah Alinia, Narjes Hendouei

**Affiliations:** a *Psychiatry and Behavioral Sciences Research Center, Addiction Institute and Department of Psychiatry, Mazandaran University of Medical Sciences, Sari, Iran. *; b *Student Research Committee, Faculty of Pharmacy, Mazandaran University of Medical Sciences, Sari, Iran.*; c *Department of Biostatistics, Health Sciences Research Center, School of Health, Mazandaran University of Medical Sciences, Sari, Iran. *; d *Department of Psychiatry, School of medicine, Babol University of Medical Sciences,Babol,Iran.*; e *Professor, Department of Internal Medicine, Diabetes Research Center, Faculty of Medicine, Mazandaran University of Medical Sciences, Sari, Iran. *; f *Department of Pharmacotherapy, Faculty of Pharmacy and Psychiatry and Behavioral Sciences Research Center, Addiction Institute, Mazandaran University of Medical Sciences, Sari, Iran.*

**Keywords:** Vitamin D deficiency, PANSS negative subscale score, PANSS positive subscale score Positive, Schizophrenic patients, Antipsychotic drugs

## Abstract

In this study, the aim was to determine whether adding vitamin D to the standard therapeutic regimen of schizophrenic male patients with inadequate vitamin D status could improve some aspects of the symptom burden or not. This study was an open parallel label randomized clinical trial. Eighty patients with chronic stable schizophrenia with residual symptoms and Vitamin D deficiency were recruited randomly and then received either 600000 IU Vitamin D injection once along with their antipsychotic regimen or with their antipsychotic regimen only. Serum vitamin D was measured twice: first at the baseline and again on the fourth month. Positive and Negative Syndrome Scale (PANSS) was assessed at the baseline and on the fourth month. During the study, the vitamin D serum changes in vitamin group and control group were 22.1 ± 19.9(95%CI = 15.9-28.8) and 0.2 ± 1.7(95%CI = 0.2-0.8) (ng/mL) (p<0.001) respectively. The changes of PANSS positive subscale score (P) were -0.1±0.7 (95%CI =-0.3-0.1) and 0.00 ± 0.8 (95%CI = -0.2-0.2) in vitamin D and control group respectively (p=0.5). The changes of PANSS negative subscale score (N) were -0.1 ± 0.7 (95%CI = -0.3-0.05) and -0.1 ± 0.5 (95%CI = -0.2-0.04) in vitamin D and control group respectively (p = 0.7) and there was a negative but not significant correlation between serum vitamin D level changes and PANSS negative subscale score (r = -0.04, p = 0.7). We did not find a relationship between serum vitamin D level changes and the improvement of negative and positive symptoms in schizophrenic patients and more randomized clinical trials are required to confirm our findings.

## Introduction

Schizophrenia is a chronic and complicated disorder with multiple mental and behavioral abnormalities. There are numerous genetic and environmental factors that may be responsible for the occurrence of schizophrenia, including maternal malnutrition especially in the first trimester, maternal exposure to influenza in the second trimester, parents’ age at the time of birth, and a range of vitamins and minerals malnutrition such as B vitamins, vitamin D, and zinc deficiencies (1). 

In addition to regulation of calcium and phosphorus homeostasis for bone health, 25-hydroxyvitamin D (vitamin D) has a significant role in brain’s growth, development, and function (2). Some epidemiological evidences suggest that vitamin D deficiency in the third trimester is a risk factor for schizophrenia, and vitamin D supplementation in the third trimester of pregnancy, infancy, and childhood can potentially improve brain development (3). Possible roles for vitamin D in the brain development are regulation of growth factors such as neuronal growth factor (NGF), synaptogenesis, and neuronal out-growth, all of which are thought to be effective in pathogenesis of schizophrenia (4). 

The enlarged lateral ventricles observed in vitamin D deficient fetuses can be reversible if vitamin D supplementation start early after the birth (5). Vitamin D fulfills its neuronal functions by binding to nuclear receptors and subsequently activating several regulators and genes, all of which tend to affect neurotrophic factors expression, cell growth, oxidative stress, autoimmune responses, and dopamine release (6). It has been shown that 1, 25 (OH)_2_ D (active form of vitamin D) nuclear receptors exist in large amounts in the pituitary gland, forebrain, hindbrain, and spinal cord of rats(7).They take part in cell homeostasis, differentiation, development of nervous system, and regulation of catecholamine levels such as dopamine (8).

Vitamin D levels have been shown to be lower in schizophrenic patients compared to healthy individuals or even depressed patients (7). There is an increasing evidence that neonates with low vitamin D levels are at higher risk for developing psychotic disorders (3). Cieslak *et al.*reported that low levels of vitamin D may play a significant role in the incidence of negative symptoms in schizophrenic patients, especially male (9). Previous studies provided evidences that indicated a higher prevalence of negative symptoms in men with schizophrenia. Also premorbid functioning and social functioning seem to be worse in males than females and substance abuse is more common in men versus women with schizophrenia (10).

 Despite the growing evidence in supporting the substantial role of vitamin D deficiency in development of schizophrenia, the effectiveness of vitamin D supplementation has not yet been addressed in improvement of symptoms of schizophrenic patients with vitamin D deficiency and their response to anti-psychotic drugs (11). In this study, we aimed to determine whether adding vitamin D to the standard therapeutic regimen of male patients with schizophrenia with inadequate vitamin D status could improve some percentage of their symptom burden.

## Materials and Methods

*Study Population*

This open parallel label randomized clinical trial (Clinical registration ID: IRCT201210163014N7), was conducted at two long-stay Psychiatric Care and Rehabilitation Centers for men, Farvardin and Iranmehr, which are Supervised by Iranian Rehabilitation Institute located in Sari in the north of Iran, between March and September of 2013. It was approved by the Ethics Committee of Mazandaran University of Medical Sciences. An informed consent was obtained from all participants’ families too.

*Patients*

During the period, from March to September of 2013, two hundred patients were registered into the trial but one hundred and twenty met the inclusion criteria. The sample size was calculated based on Itzhaky *et al*. study (12). The inclusion criteria were patients aged between 18-65 years and met DSM-IV-TR (13) criteria for schizophrenia with baseline serum vitamin D concentrations less than 30 ng/mL (vitamin D deficiency and insufficiency have been defined as 25(OH) D of less than 20 ng/mL and 21–29 ng/mL, respectively) (14), BMI of 16- 25 Kg/m^2^ and if they were suffering from the disease for more than 3 years with residual symptomatology despite antipsychotic treatment. All patients were on antipsychotics for at least 1 year and a stable dose for at least 2 month were included in the trial and the antipsychotics dose remained unchanged during the trial. Patients who met the inclusion criteria were included in the study and randomized into 2 treatment groups using the rand between logs after giving numbers to the patients in Excel software.

The subjects were excluded if they had renal and hepatic failure or parathyroid disorder before or during the study; patients who used phosphor, calcium or vitamin D supplements, or teriparatide or had co-administered medications of anticonvulsants, ketoconazole and corticosteroids and had history of other psychiatric and neurologic disorders were excluded too. 

The patients who received two intramuscular D_3_ injections (2-mL ampoule, 300 000 IU/mL in sesame oil, made by Iran hormone, Iran) at baseline along with their antipsychotic regimen and followed for three months (15-17) were known as the intervention group. The patients in the control group received their antipsychotic regimen only and followed for three months. At the end of the study, all the patients in control group were treated with vitamin D supplement; however, they did not receive any follow up. 

All schizophrenic patients were managed based on the guidelines of the American Psychiatric Association (18). The diagnosis was made by two psychiatrists independently, according to the DSM-IV-TR criteria (13). The patients were prescribed antipsychotics (on average 342 ± 15.6 mg chlorpromazine equivalents/day) at the time of blood sampling. During the study period, only co- medication with anticholinergic drugs and benzodiazepines were allowed. Most of the subjects suffered from residual, paranoid, and undifferentiated schizophrenia respectively. During the study, we tried to keep the diet containing vitamin D and the amount of sunlight exposure the same for all of the patients. Two psychiatric and rehabilitation centers were 20 Km apart with the same height above sea level and the same weather in Mazandaran province. The study was conducted in spring and summer (March to September of 2013). The patients were allowed to attend the outdoors, two hours in the morning and two hours in the afternoon in both psychiatric and rehabilitation centers. The diet of patients was similar in terms of foods containing vitamin D like fish, oils, meat and eggs. 

*Sample collection and measurement*

A sample of 3 mL of venous blood was collected into a test tube that did not contain anticoagulant at the baseline by the end of the third month (one month after the last injection) (19). Each sample was centrifuged (3500×g) for 15 min. Then the serum was separated and the serum vitamin D concentration was analyzed using a commercially available 25-OH Vitamin D ELISA kit (EUROIMMUN, Medizinische Labordiagnostika AG, UK) according to the manufacturer’s instructions. This ELISA kit is designed for the in vitro determination of 25-OH vitamin D in human serum or plasma samples.

For each patient, a set of variables were collected including demographics (age, gender, marital and smoking status), subtypes of schizophrenia, duration of disease, and initial Positive and Negative Syndrome Scale score (PANSS) (20) .

Serum creatinine, blood urea nitrogen (BUN), alanine transaminase (ALT), aspartate aminotransferase (AST), glucose, hematocrit, hemoglobin, platelet, and white blood cell (WBC) count were only measured at the baseline except for serum calcium and phosphorus levels which were measured at the baseline and then monthly for three months to prevent vitamin D toxicity (17, 19). PANSS was assessed at the baseline and on the first and fourth months (at the end of third month). The PANSS assessment was blind and made by two trained and expert psychologists independently. Also serum vitamin D level was assessed at the baseline and on the fourth month (16). Serum parathyroid hormone level for all the patients was checked at the baseline too.

*Statistical analysis*

All data were assessed for normality by “one sample Kolmogorov-Smirnov” test. Qualitative variables were recorded according to frequency and percentage and quantitative variables in terms of Mean ± SD (Standard Deviation). To compare continuous variables in two groups, we used independent sample t-test and to compare categorical variables we used either the Chi-Square or Fisher›s exact test. Kruskal-Wallis test was used, for comparing quantitative variables in different antipsychotic drug groups. Repeated measurement analysis was conducted for serial comparisons of positive and negative subscale symptoms and comparisons between groups in different times of treatment; pairwise comparison was applied by Scheffe. The correlation between serum vitamin D level and negative and positive symptoms at baseline was made by Pearson correlation test. All statistical analyses were conducted using the software SPSS version 15 (SPSS Inc., Chicago, IL, USA) and P values of less than 0.05 were considered significant.

## Results

**Figure 1:**
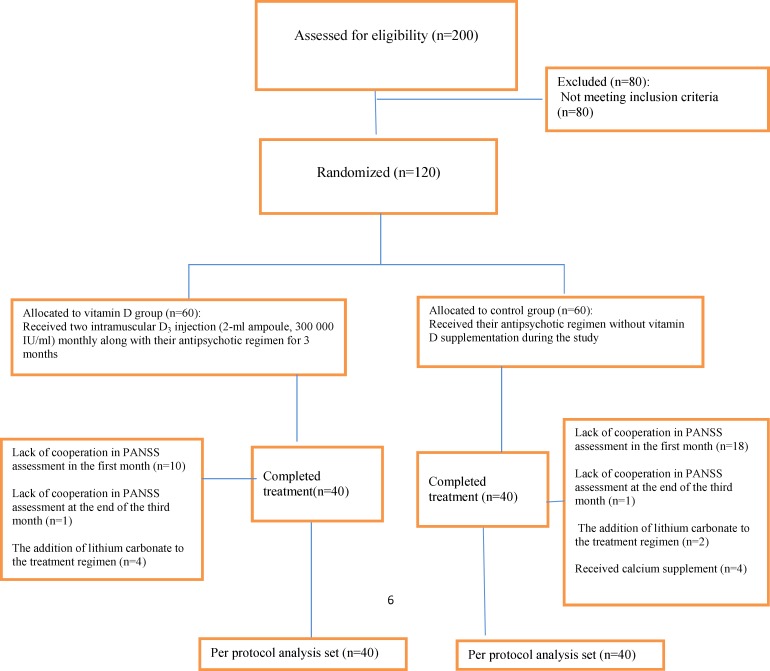
Flow diagram of enrolment, randomization and follow-up

One hundred and twenty patients who met the inclusion criteria entered the study (Figure 1).

Ten patients in vitamin D group and 18 in control group at the end of the first month, and 4 patients in vitamin D and 1 in control group on the fourth month did not cooperate in PANSS assessment. Lithium carbonate was added to the treatment regimen of 2 patients in control group and 1 in vitamin D, due to the recursion of the symptoms after 20 days after starting the study. Four patients in the control group were excluded because of receiving calcium supplements two weeks after receiving vitamin D. Finally, eighty patients completed the study in which 40 of them received vitamin D supplement (intervention group), and 40 patients did not (control group). Baseline demographics and clinical characteristics were shown in Table 1. There was no significant difference between two patient groups regarding serum parathyroid hormone which was within the normal range in all the patients at the baseline. There was no significant difference between the groups in prescribed antipsychotic drugs. Prescribed drugs for all the patients with schizophrenia during the study were shown in Table 1.

**Table 1:** Baseline demographic and clinical measurements in schizophrenic patients

	Vitamin D group (n=40)		Control group (n=40)		P Value
Age (y)	46.23 ± 10.7 (95%CI =42.76-49.7)		48.17 ± 11.06 (95%CI =44.72-51.61)		0.427^a^
Marital status (n)					
- Single - Married - Divorce	27 4 9		20 8 22		0. 7 a 0.257^b^ 0. 5 ^b^
Smoking( n )	22		26		0.528 ^b^
Gender( n)					
Male	40		40		0.96 ^b^
Duration of disease(y)	6.3 ± 3.2		6.2 ± 3.3		0.91
Subtypes of schizophrenia( n)					
Paranoid Residual Undiffrential	4 32 4		3 35 2		0.1 ^b^ 0.4 ^b^ 0.27 ^b^
Vitamin D concentration(ng/ml)	14.7 ±6.001 (95%CI =12.86-16.75)		14.2 ± 6.97997 (95%CI =11.9265-16.5)		0.2 a
PANSS positive subscale score	12.85 ± 6.904 (95%CI =10.6-15.06)		11.43 ± 5.429 (95%CI =9.7-13.1)		0.3 a
PANSS negative subscale score	20.33 ± 10.7 (95%CI =16.9-23.7)		17.8 ± 9.8 (95%CI =14.9-21.08)		0.341 a
The prescribed antipsychotics during the study n (%)					
Risperidone		26(65)		24 (60)	0.8^c^
Olanzapine		3 (7.5)		4(10)	0.8 ^c^
Fuphenazinedecanoate		0 (0)		1 (2.5)	0.95 ^c^
Olanzapine &Fluphenazinedecanoate		3 (7.5)		2 (5)	0.83 ^c^
Risperidone&Fluphenazinedecanoate		8 (20)		9 (22.5)	0.9 ^c^
Antipsychotic dosage (Chlorpromazine equivalents)		350 ± 20.8		335 ± 10.5	0.9 a

PANSS: Positive and Negative Syndrome Scale

* p < 0.05 considered significant

a : Independent Samples Test,

b : Chi-Square Tests ,

c : Fisher Exact Test

**Table 2:** Vitamin D concentration at baseline and on the fourth month in all schizophrenic patients during the study

Vitamin D status	Baseline(n:40)			on fourth month(n:40)		
	Vitamin D	Control	P value	Vitamin D	Control	P value
Vitamin D concentration (ng/mL)	14.7 ±6.001 (95%CI =12.86-16.75)	14.2 ± 6.97997 (95%CI =11.9265-16.5)	0. 7^a^	50.5±9.04 (95%CI =46.6-54.4)	13.9914±6.8 (95%CI =11.7213-16.2)	<0.001^a^ *
Deficiency (<20ng/mL) (n)	33(82.5%)	31(77.5%)		-	29(72.5%)	
Insufficiency (20-30ng/mL) (n)	7(17.5%)	9(22.5%)		2(5%)	11(27.5%)	
Normal (30-50ng/mL) (n)	-	-		30(75%)	-	
Upper normal (50-70ng/mL) (n)	-	-		8(20%)	-	

* p < 0.05 considered significant

a Independent Samples Test

**Table 3:** PANSS subscale scores in schizophrenic patients during the study

	Intervention group	Control group
**P**_1_ **P**_2_ **P**_3_	12.85 ± 6.904 (95%CI =10.6-15.06) 12.65 ± 6.7 (95%CI =10.4-14.8) 12.73 ± 6.7 (95%CI =10.5-14.8)	11.43 ± 5.429 (95%CI =9.7-13.1) 11.40 ± 5.4 (95%CI =9.7-13.09) 11.36 ± 5.3 (95%CI =9.6-13.06)
**N**_1_ **N**_2_ **N**_3_	20.33 ± 10.7 (95%CI =16.9-23.7) 20.23 ± 10.6 (95%CI =16.8-23.6) 20.15 ± 10.7 (95%CI =16.7-23.5)	17.8 ± 9.8 (95%CI =14.9-21.08) 17.88 ± 9.8 (95%CI =14.8-20.9) 17.88 ± 9.8 (95%CI =14.8-20.9)

-P_1_: PANSS positive subscale score at baseline,

P_2_: PANSS positive subscale score on the first month,

P_3_: PANSS positive subscale score on the 4 months ,

N_1_: PANSS negative subscale score at baseline ,

N_2_: PANSS negative subscale score on the first month ,

N_3_: PANSS negative subscale score on the 4 month

-Repeated Measurement analysis

* p < 0.05 considered significant

**Table 4:** Correlation between Vitamin D concentration changes and PANSS positive and negative subscale scores changes in schizophrenic patients in the intervention group

	**N**_ diff_	**P**_diff_
**Vitamin D concentration changes (ng/ml)**	r = - 0.04 P = 0.7	r = 0.1 P = 0.4

Pearson correlation

**N**_ diff_ :PANSS negative subscale scores changes, **P**_diff_ :PANSS positive subscale scores changes

* p < 0.05 considered significant

The mean serum concentrations of vitamin D were 14.7 ± 6.001 (95%CI = 12.86-16.75) (ng/mL) and 14.2 ± 6.97997 (95%CI = 11.9-16.5) (ng/mL) for intervention and control groups, respectively (p = 0. 2). During the study, vitamin D concentration increased significantly in vitamin D group (*p *= 0.03) and the vitamin D serum changes in vitamin group and control group were significant (p<0.001). Serum vitamin D level in patients receiving different antipsychotic drugs had a significant difference at the beginning of the study (p<0.001). In serum vitamin D level, this difference was significant in patients receiving risperidone with patients treated by Olanzapine and Fluphenazine decanoate combination and Risperidone and Fluphenazine decanoate combination (p = 0.003 and p = 0.008, respectively). But at the end of the study no differences were reported about the serum vitamin D level in patients receiving different antipsychotic drugs (p = 0.1). 

Vitamin D concentration at baseline and on the fourth month in all schizophrenic patients during the study was shown in Table 2.

At the baseline, PANSS positive subscale score and PANSS negative subscale score were not significantly different between the groups (p = 0.5 and p = 0.3, respectively). During the study, PANSS negative subscale score and PANSS positive subscale score decreased in both groups but this reduction was not significant (p = 0.5,p = 0.7), and there were no differences between groups (p = 0.3,p = 0.3) (Table 3).

The changes of PANSS positive subscale score were -0.1 ± 0.7 (95%CI = -0.3-0.1) and 0.00 ± 0.8 (95%CI = -0.2-0.2) in vitamin D and control group respectively (p = 0.5). The changes of PANSS negative subscale score was -0.1 ± 0.7 (95%CI = -0.3-0.05) and -0.1 ± 0.5 (95%CI = -0.2-0.04) in vitamin D and control group respectively (p = 0.7).

The correlation between changes of Vitamin D concentrations with PANSS negative subscale score and PANSS positive subscale score in vitamin D group are illustrated in Table 4.

During the study, any side effects observed in schizophrenic patients were treated with vitamin D.

## Discussion

Supplementation with vitamin D in other chronic diseases such as cardiovascular, kidney, and autoimmune diseases have been documented by other researchers (21). However, to the best of our knowledge, this study is the first one to assess the effect of adding vitamin D supplement to the diet of patients with chronic schizophrenia on the improvement of their residual symptoms (22). 

In our study, we found a negative but not significant correlation between serum vitamin D level changes and PANSS negative subscale score in intervention group.Althought we did not find a relationship between serum vitamin D level changes and the improvement of negative and positive symptoms in schizophrenic patients and more randomized clinical trials are required to confirm our findings.

Vitamin D is categorized into neuroactive steroid groups due to association of its deficiency to several neuropsychiatric disorders, such as Alzheimer’s disease, autism, depression, and schizophrenia(3). Three main forms of vitamin D3 (25(OH) D3, 1, 25(OH)2D3, and 24, 25(OH)2D3) have been found in human cerebrospinal fluid(23); This reinforces to believe that vitamin D could possibly have vital functions in the brain. Moreover, maternal vitamin D deficiency has been linked to schizophrenia in neonates (3), and many studies have shown that vitamin D deficiency during pregnancy and early life impedes brain growth and development in certain regions of brain(24). McGrath *et al*. in a 31-year cohort study evaluated the role of vitamin D deficiency in the first year of life and the risk of schizophrenia in adulthood. The results of this study showed that the use of vitamin D supplements (either irregular or regular) in the first year of life was associated with a reduced risk of schizophrenia, especially in men. In this study, the minimum effective dose of vitamin D was 2000 IU to reduce the risk of schizophrenia (25).

In recent years, there were several reports about vitamin D deficiency in schizophrenia patients both in acute (7) and chronic phase(8, 12, 26); and in some cases it was considered as a risk factor in relapsing of schizophrenia; however, it is not clear whether vitamin D deficiency is due to the schizophrenia or vitamin D deficiency is caused by this illness. 

Data show that there are a significant overlap between genes involved in schizophrenia and the ones related to vitamin D synthesis. So, it can be assumed that schizophrenia occurrence and vitamin D deficiency have a genetic co-occurrence(22). Current studies showed that in male schizophrenic patients, hypovitaminosis D has associations to an increase of negative symptoms and a decrease in functioning, and it is associated with lesser hallucinatory behavior and emotional withdrawal, but increased anti-social aggression in female schizophrenic patients(9).The data obtained from studies assessing the relationship between serum vitamin D level and the severity of psychotic symptoms is contradictory. Results achieved from Berg *et al*. (22) and Cieslak *et al*.(9) showed an inverse relationship between serum vitamin D level and psychomotor activity, physical energy, and the severity of negative symptoms; however, in Itzhaky *et al.* study similar to the present study (12) there were no relationships between serum vitamin D level and severity of illness according to PANSS. 

It seems that the difference in the severity of positive and negative symptoms, the difference in the sample size, baseline serum vitamin D, the illness duration, ethnicity, sun exposure, nutrition status, skin color, study location, the cutoff point of vitamin D deficiency, vitamin D assessment method, and hospitalization status (in-patient or outpatient) are the most important factors affecting different results in these studies. Our findings on mean serum vitamin D levels at baseline were similar to a recently published Yuksel *et al.* (22) in schizophrenic patients in remission phase in this regard.In the present study, we did not find a relationship between serum vitamin D level changes and the improvement of negative and positive symptoms in schizophrenic patients.Graham *et al*. (27) and Cieslak *et al*. (9) reported a reverse and strong relationship between vitamin D insufficiency and negative symptoms in schizophrenia, schizoaffective, and schizophreniform patients; however, in a comparison to the present study, the severity of negative symptoms in patients was lower, and the sample size was less. Also, the serum vitamin D level in patients was much higher than the one in our study. 

One of the strengths of this study was controlling factors affecting vitamin D deficiency such as smoking status, ethnicity, skin color, study location, and BMI in patients not considered in previous studies (28). In addition, it was tried that the diet containing vitamin D and the amount of sunlight exposure for all the patients were the same during the study. 

The results in present study were similar to the Yuksel *et al*. (22). They reported; the severity of negative and positive symptoms in schizophrenic patients in remission phase is higher in patients with lower vitamin D levels. But all the patients in their study were outpatients and 60% of patients in remission phase, received sufficient intake of vitamin and 51% had more than 45 minute per week sunlight exposure. 

One of the other strengths in this research was the study of the effect of antipsychotic drugs on the serum vitamin D level. Lauth *et al*. (29) showed that antipsychotic drugs with modulation of enzyme level 7-Dehydrocholesterol Reductase can affect the production of the vitamin D in vitro. Atypical antipsychotic drugs can cause a decrease in vitamin D level by an increase in patients’ BMI, too (30).

In the present study, the patients have been under the treatment with the fixed dose of atypical antipsychotic drugs at least for two months which is considered a suitable time for affecting the drugs on the serum vitamin D level via BMI increasing. The results showed that there were significant differences between the types of drugs and vitamin D level in baseline in all patients but we found no differences between antipsychotic groups in vitamin D levels at the end of the study. More studies are needed in order to assess the effect of antipsychotic drugs on the serum vitamin D level in schizophrenic patients.

This study has some limitations which have to be pointed out. The same vitamin D_3_ doses in deficient and insufficient patients were used, but dosing of vitamin D_3_ depends upon the nature and severity of the deficiency. The patients with deficiency require a loading dose (50,000 IU of vitamin D orally once weekly for 2-3 months, or 3 times weekly for 1 month) and for the patients with mild to moderate deficiency a shorter treatment interval or lower dose (11-25 ng/mL) may be effective(17). However, a review of multiple loading algorithms suggested that a minimum total dose of 600,000 IU best predicted an end-of-treatment 25(OH) D level greater than 30 ng/mL(17). In our study, we prescribed a total dose of 600,000 IU (two intramuscular D_3_ injection, 2-mL ampoule, 300 000 IU/mL) for patients in intervention group due to lack of appropriate compliance to weekly or daily oral treatment regimens in schizophrenic patients. 

Also, one of the limitations of this study is having no assessments of the general sub score and at last total PANSS. 

Our study paves the way for designing larger clinical trials to address unanswered questions such as proper vitamin D serum levels to obtain maximum beneficial effects and vitamin D dosing regimens to achieve desired serum levels (10). We recommend that various studies be designed with different doses of vitamin D based on serum vitamin D with longer periods of treatment with more schizophrenic patients under different anti-psychotic regimens. Also, determining the effectiveness of vitamin D in reducing the symptoms of schizophrenic patients with no background of vitamin D deficiency is an interesting question to be addressed in future studies.

## Conclusion

In our study, we found a negative but not significant correlation between serum vitamin D level changes and PANSS negative subscale score in intervention group.Althought we did not find a relationship between serum vitamin D level changes and the improvement of negative and positive symptoms in schizophrenic patients, more randomized clinical trials are required to confirm our findings.
